# Sphingomyelin is a prospective metabolic immune checkpoint for natural killer cells

**DOI:** 10.1002/ctm2.1395

**Published:** 2023-08-30

**Authors:** Hongdi Ma, Xuben Wang, Xiaohu Zheng, Haiming Wei

**Affiliations:** ^1^ Hefei National Research Center for Physical Sciences at Microscale The CAS Key Laboratory of Innate Immunity and Chronic Disease School of Basic Medical Sciences Center for Advanced Interdisciplinary Science and Biomedicine of IHM Division of Life Sciences and Medicine University of Science and Technology of China Hefei China; ^2^ Institute of Immunology University of Science and Technology of China Hefei China; ^3^ Key Laboratory of Quantitative Synthetic Biology Shenzhen Institute of Synthetic Biology Shenzhen Institute of Advanced Technology Chinese Academy of Sciences Shenzhen China

**Keywords:** metabolic immune checkpoint, membrane protrusions, NK cells, Sphingomyelin

Lymphocyte surface checkpoint blockade has revolutionized cancer treatment. However, robust responses to immune checkpoint inhibitors (ICI) are observed only in a subset of treated patients, and a significant fraction fails to achieve sustained therapeutic responses from these interventions.^1^ Consequently, a key challenge in tumour immunotherapy lies in comprehending the mechanisms of non‐responsiveness to ICI, with the aim of augmenting the proportion of patients who derive benefits from this therapeutic approach.

## 1 KILLER CELLS HINDER TUMOUR CELL RECOGNITION AND LYTIC IMMUNOLOGICAL SYNAPSE INITIATION

**FIGURE 1:**
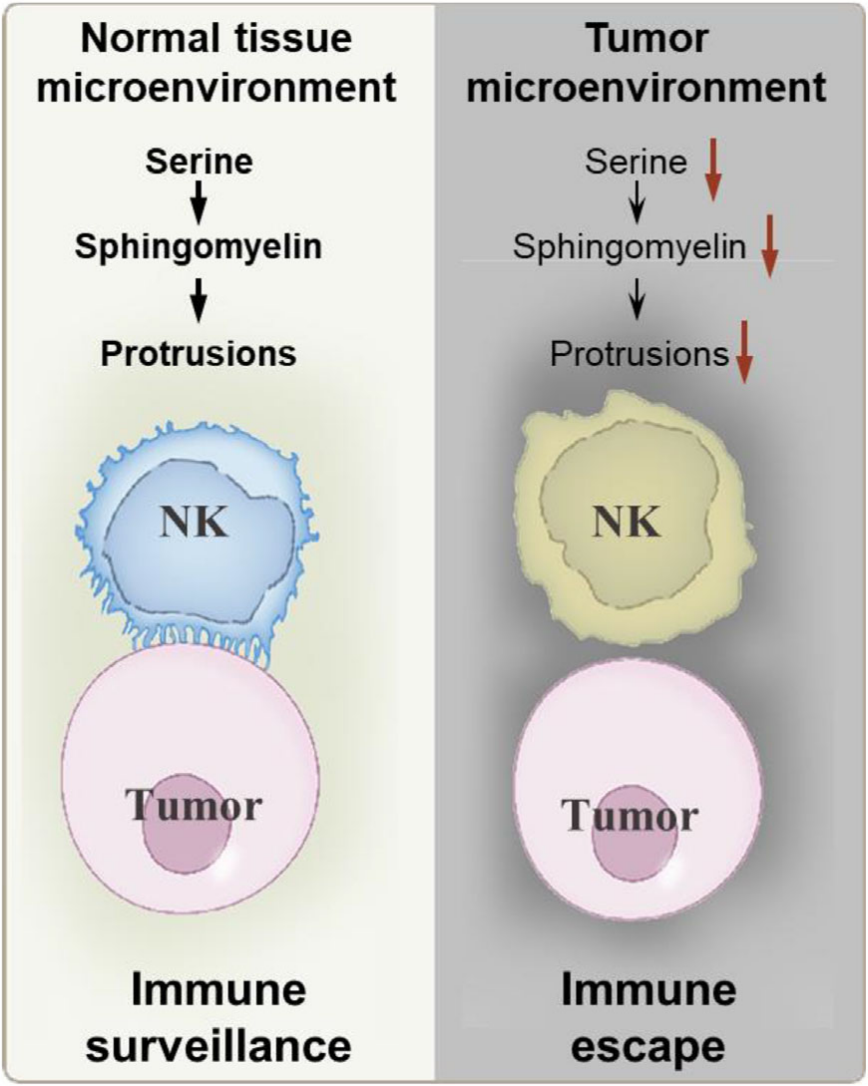
Comparison of natural killer (NK) cell membrane surface features between the normal tissue microenvironment and the tumour microenvironment. NK cells within the normal tissue exhibit plentiful membrane protrusions, while those in the tumour microenvironment display reduced protrusions. This deficiency in membrane protrusions hampers the capacity of tumour microenvironment NK cells to effectively recognize tumour cells and establish lytic immune synapses. The disruption in serine‐sphingomyelin metabolism contributes to the loss of NK cell membrane protrusions within the tumour microenvironment.

Cytotoxicity induction by natural killer (NK) cells necessitates their recognition and contact with target cells (tumour cells), culminating in the establishment of lytic immune synapses.^2^ Recently, we elucidated that normal NK cells, including those from the liver and peripheral sources, showcase abundant membrane surface protrusions. These protrusions, polarized towards tumour cells, constitute distinct lytic immune synapses.^3^ However, intratumoral NK cells, hampered by disrupted membrane protrusions, are impeded in recognizing and establishing lytic immunological synapses with tumour cells, leading to impaired tumour cell killing.^3^ These membrane surface protrusions akin to NK cell ‘tactile organs’ facilitate effective tumour cell interaction. In the tumour microenvironment, the absence of this pivotal feature compromises the ability of NK cells to locate and engage tumour cells. Even when a fraction of intratumoral NK cells manage target recognition, the dearth of functional membrane protrusions hampers the formation of mature lytic synapses (Figure 1). Importantly, our findings extend beyond liver cancer, encompassing lung, colon, and ovarian cancers.^3^ Altogether, this unveils a novel perspective on a prominent mechanism enabling tumour evasion from NK cell‐mediated killing.

## 2 SPHINGOMYELINASE IS A PROSPECTIVE VULNERABLE "METABOLIC IMMUNE CHECKPOINT"

Within the tumour microenvironment, nutritional and metabolic competition among various cell types is apparent.^4^ This often results in tumour cells having the upper hand, leaving immune cells at a disadvantage and thereby restricting their access to essential nutrients. Research into intratumoral T cell metabolism underscores how glucose scarcity, orchestrated by tumors, reshapes metabolic pathways, leading to diminished effector function. Our own investigations revealed a dependence of NK cell membrane protrusion formation on sphingomyelin synthesis.^3^ In the context of the tumour microenvironment, the insufficient availability of serine hampers both sphingomyelin synthesis and the formation of NK cell membrane protrusions^1^ (Figure 1).

From the burgeoning field of immuno‐metabolism emerges a compelling strategy that could reinvigorate anti‐tumour immune responses. Recent studies have highlighted established metabolic regulators such as Glut, mTOR and PDHK1 as pivotal checkpoints in T cell immunity against tumors.^5^ Employing sphingomyelin catabolism inhibitors, including the acidic SMase inhibitor (A‐SMase inhibitor, LCL521) and the neutral SMase inhibitor (N‐SMase inhibitor, GW4869), we successfully reinstated the tumour cell lysis capacity of intratumoral NK cells.^3^ A noteworthy conceptual implication is that the sphingomyelin catabolism enzyme, sphingomyelinase, warrants recognition as a “metabolic immune checkpoint.”

## 3 ENHANCED ANTI‐TUMOR EFFECTS THROUGH DUAL BLOCKADE OF SPHINGOMYELINASE CATABOLISM AND IMMUNE CHECKPOINTS

The varying response rates among patients to immunotherapies, including checkpoint blockade, are now widely acknowledged, prompting extensive research into the multifaceted underpinnings of these disparate outcomes. The precise stage at which tumour progression triggers disruptions in infiltrated NK cell membrane protrusions remains undetermined. In individuals, infiltrating NK cells can exhibit both disrupted membrane protrusions and heightened expression of inhibitory immune checkpoint molecules. Notably, a compelling avenue emerges with the combination therapy involving concurrent blockade of sphingomyelinase catabolism and immune checkpoints. Our findings illustrate that compared to monotherapies, dual blockade combinations encompassing GW4869 (N‐SMase inhibitor) combined with a Tim3‐blocking antibody substantially amplify the tumour lysis capabilities of intratumoral NK cells.^3^ Consequently, SMase inhibitors hold promise as synergistic agents in conjunction with immune checkpoint protein blockade.

## CONFLICT OF INTEREST STATEMENT

The authors declare no conflict of interest.
